# Optimization of Mass Spectrometric Parameters in Data Dependent Acquisition for Untargeted Metabolomics on the Basis of Putative Assignments

**DOI:** 10.1021/jasms.3c00084

**Published:** 2023-07-07

**Authors:** Hailemariam Abrha Assress, Mario G. Ferruzzi, Renny S. Lan

**Affiliations:** † Arkansas Children’s Nutrition Center, Little Rock, Arkansas 72202, United States; ‡ Department of Pediatrics, University of Arkansas for Medical Sciences, Little Rock, Arkansas 72205, United States

**Keywords:** Optimization, Mass spectrometric parameters, Data dependent acquisition, Untargeted metabolomics, Orbitrap mass spectrometer

## Abstract

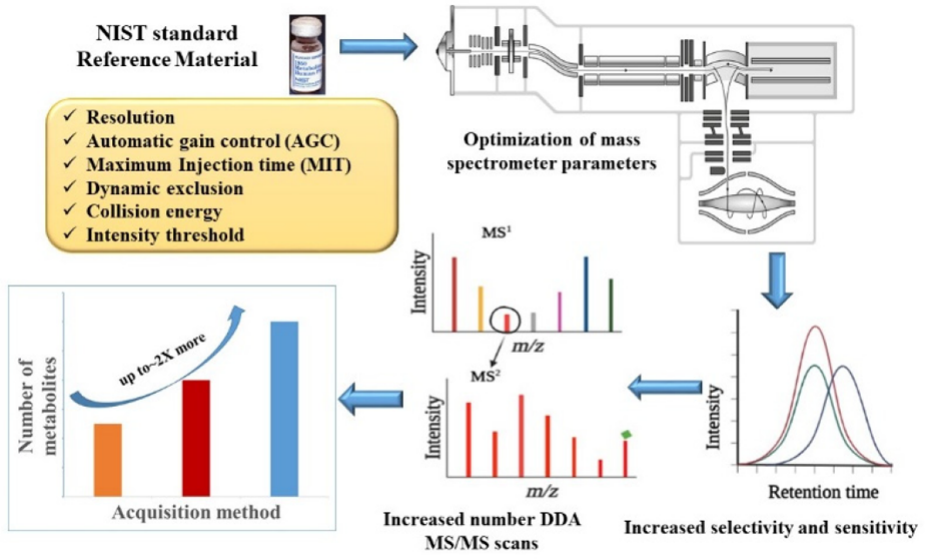

Optimization of mass spectrometric parameters for a data dependent acquisition (DDA) experiment is essential to increase the MS/MS coverage and hence increase metabolite identifications in untargeted metabolomics. We explored the influence of mass spectrometric parameters including mass resolution, radio frequency (RF) level, signal intensity threshold, number of MS/MS events, cycle time, collision energy, maximum ion injection time (MIT), dynamic exclusion, and automatic gain control (AGC) target value on metabolite annotations on an Exploris 480-Orbitrap mass spectrometer. Optimal annotation results were obtained by performing ten data dependent MS/MS scans with a mass isolation window of 2.0 *m*/*z* and a minimum signal intensity threshold of 1 × 10^4^ at a mass resolution of 180,000 for MS and 30,000 for MS/MS, while maintaining the RF level at 70%. Furthermore, combining an AGC target value of 5 × 10^6^ and MIT of 100 ms for MS and an AGC target value of 1 × 10^5^ and an MIT of 50 ms for MS/MS scans provided an improved number of annotated metabolites. A 10 s exclusion duration and a two stepped collision energy were optimal for higher spectral quality. These findings confirm that MS parameters do influence metabolomics results, and propose strategies for increasing metabolite coverage in untargeted metabolomics. A limitation of this work is that our parameters were only optimized for one RPLC method on single matrix and may be different for other protocols. Additionally, no metabolites were identified at level 1 confidence. The results presented here are based on metabolite annotations and need to be validated with authentic standards.

## Introduction

Mass spectrometry (MS) has evolved as the preferred analytical method for proteomics, lipidomics and metabolomics.^1^ Particularly, MS has been used in both untargeted and targeted metabolomics research approaches, allowing thousands of biologically active metabolites to be identified and quantified at trace levels in a wide range of matrices.^2^ Currently, MS-based metabolomics platforms and workflows are leveraged in areas of drug discovery,^3^ toxicology,^4^ biomarker discovery,^5^ precision medicine,^6^ prevention and diagnosis of human diseases,^7^ microbial biotechnology,^8^ plant biotechnology,^9^ exposome research,^10^ and food and nutrition research^11^ and in the investigation of contaminants of emerging concern (CECs).^12^

MS instrumentation has experienced several levels of major improvements in mass analyzer technology and instrument layout that have also enabled it to rapidly expand analytical power and application range.^13,14^ Significant advances in ionization, separation, and data processing technologies have contributed to broader application ranges and capacity.^15^ The increasing shift to high-resolution accurate-mass (HRAM) analysis has been one of the major themes of the previous two decades of innovation.^13,16^ Orbitrap mass spectrometry has become one of the major drivers and beneficiaries of this transition. Over the years, Orbitrap designs and capabilities have grown dramatically in numerous aspects.^17^ The recently introduced Orbitrap Exploris 480, a hybrid quadrupole-Orbitrap MS instrument, is capable of providing high quality high energy collisional dissociation (HCD) mass spectra with a resolving powers from 7500 to 480,000 at *m*/*z* 200.^18,19^ The increased scan speed, high resolution, improved sensitivity and robustness of the instrument has made it popular choice in proteomics and untargeted metabolomics research.^19,20^

When metabolites are extracted from biological materials and separated using UHPLC before introduction into the MS instrument, tens of thousands of signals are typically detected in an untargeted metabolomics experiment.^21^ The majority of metabolomics data sets from the MS are generated using the data-dependent acquisition (DDA) technique, which involves the mass spectrometer alternating between a survey scan (MS1) and a series of data-dependent tandem MS scans (MS/MS).^22,23^ During data acquisition, the MS instrument looks for metabolite precursor signals in each MS1 spectrum. Then, MS/MS spectra are generated by selecting high abundant precursors for fragmentation up on meeting predetermined signal intensity.^24^ Metabolites are then identified by matching the acquired MS/MS spectra to an online database or in-house libraries. There are several mass spectrometric parameters in DDA that influence the quality and the quantity of MS/MS spectra collected, which in turn influence the metabolite identification in untargeted metabolomic analysis.^25^ The success of untargeted metabolomics depends not only on the instrument performance but also on the optimization of the mass spectrometric parameters. Therefore, optimization of the parameters for DDA experiment is essential to increase the MS/MS coverage and hence increase rate of identification in untargeted metabolomics.^26^ The published literature contains large discrepancies in the use of the mass spectrometric parameters for untargeted metabolomic analysis.^27,28^ Furthermore, essential MS parameters required to replicate an experiment have been omitted in a notable proportion of publications. It can be challenging for metabolomics researchers to choose which parameter to use for their analyses due to the heterogeneity and sometimes even the lack of descriptions of instrument settings. This work focuses on the evaluation and optimization of MS parameters on the Orbitrap Exploris 480 mass spectrometer for improved metabolite coverage using DDA based untargeted metabolomics.

## Experimental Section

### Materials and Reagents

LC-MS optima grade water, methanol, acetonitrile, and formic acid were purchased from Thermo Fisher Scientific (Waltham, MA). Standard reference material (SRM) 1950 serum was purchased from the National Institute of Standards and Technology (NIST) (Gaithersburg, MD).

### Extraction of Metabolites

NIST SRM 1950 reference human plasma was extracted by using an in-house methanol extraction method. Cold methanol (800 μL) was added to 200 μL of frozen plasma in a 1.7 mL centrifuge tube. The mixture was incubated for 15 min at 4 °C on a ThermoMixer (Eppendorf Inc., Enfield, CT) and then centrifuged (18,000*g*) at 4 °C for 10 min (Centrifuge 5430R, Eppendorf Inc., Enfield, CT). The supernatant was divided into 100 μL aliquots, each dried by using a vacuum concentrator (SpeedVac SPD210, Thermo Fisher Scientific Waltham, MA). Extracts were then reconstituted in 200 μL of water/methanol (95:5) modified with 0.1% formic acid. Dried plasma extracts were stored at −80 °C until analysis.

### Chromatography and Mass Spectrometry

Instrumental analysis was performed on a Vanquish UHPLC coupled to an Orbitrap Exploris 480 mass spectrometer (ThermoFisher Scientific, Waltham, MA) equipped with high flow and low flow heat-electrospray ionization (HESI) probes. Chromatographic separations were performed using Acquity Premier CSH C18 1.7 μm × 2.1 × 100 mm Column (Waters, USA) at a flow rate of 0.3 mL min^–1^. The mobile phase system consisted of water (A) and acetonitrile (B), both acidified with 0.1% of formic acid, using the following gradient elution: 0 min, 0% B; 2 min, 40% B; 8 min, 98% B; 10 min, 98% B; 10.5 min, 0% B; 15 min, 0% B. A column temperature of 40 °C and injection volume of 5.0 μL were used during the analysis.

The global settings for the MS were as follows: the instrument was operated in a positive mode with a positive ion spray voltage of 3.6 kV. Sheath gas, auxiliary gas, and sweep gas were set at 35, 10, and 1 arbitrary units (Arb), respectively, while both the ion transfer tube (ITT) temperature and vaporization temperature were set at 350 °C. Full scan MS spectra in triplicate and one MS/MS spectra were recorded in the range of 50–750 *m*/*z* in a DDA mode for each parameter setting. Other MS operating parameters including resolution, RF level, intensity threshold, mass isolation width, number of microscans, number of data dependent scans (TopN), dynamic exclusion, maximum injection time (MIT) and automatic gain control (AGC) were optimized using the one factor at a time (OFAT) approach.^29^ Initially, full MS spectra were acquired at a resolution of 30k at 200 *m*/*z*, a standard AGC (where the system sets the recommended target in an automated fashion), RF level of 60% and a maximum injection time of 100 ms. For the MS/MS, a standard AGC, a stepped HCD collision energy of 20, 40, and 60, maximum injection time of 50 ms, a resolution of 30k, and a mass isolation width of 2 *m*/*z* were used. The Top 5 MS/MS scans were recorded from signals above the threshold of 100,000. Both the full scan and the MS/MS spectra were acquired in profile mode. The significance of the full MS and MS/MS operating parameters and their influence on the coverage of metabolite was evaluated. After a parameter has been evaluated, the optimization tests on other parameters continued with that parameter’s optimal value. The tested values for all the parameters optimized are summarized in Table 1. Description of how each parameter influences data acquisition and hence metabolite coverage is included in the Results and Discussion section. The mass spectrometer calibration in the low mass and high mass range was performed with the Pierce FlexMix calibration (ThermoFisher Scientific, Waltham, MA).

**Table 1:** Instrument Parameters Optimized and Values Tested for Each Parameter

parameter optimized	values tested for full scan	values tested for ddMS/MS
resolution	30k, 60k, 120k, 180k, 240k, and 480k	30k, 45k, 60k, and 120k
RF lens (%)	10, 20, 30, 40, 50, 60, 70, 80, 90, 100	N/A
intensity threshold	N/A	1e,^3^ 1e,^4^ 1e,^5^ 1e,^6^ 1e,^7^ and 1e^8^
mass isolation width (*m*/*z*)	N/A	0.4, 0.8, 1.2, 1.6, 2, 2.4, 2.8, 3.2, 3.6, 4, 4.4, 4.8, 5.2, 5.6, and 6
microscan	1, 2, 3, 4, 5	1, 2, 3, 4, and 5
top N	N/A	5, 8, 10, 12, 15, and 20
cycle time (s)	N/A	1, 3, 5, and 7
automatic gain control (AGC) in %	standard,^a^ 100, 200, 300, 400, 500, 1000	50, 100, 200, 300, 400, and 500
maximum ion injection (MIT) in ms	auto,^b^ 25, 50, 75, 100, 125, 150, 200, 250, 300	50, 100, 150, 200, 250, and 300
dynamic exclusion	N/A	Repeat count 1: exclusion duration 3, 5, 7, 10, 15, 20, 40, 60, 80, and 100 s
		Repeat count 2: repeat duration 30 s; exclusion duration 20, 40, 50, 60, 80, and 100 s
collision energy (CE)	N/A	Fixed CE: 20, 30, 40, 50, 60, 80, and 90
		Stepped two CE: 10&30, 10&40, 10&50, 10&60, 20&30, 20&40, 20&60, 20&80, 30&40, 30&50, 30&60, 30&80, 40&50, 40&60, 40&80, 50&60, 50&70
		Stepped three CE: 10, 30, and 50; 20, 30, and 40; 20, 40, and 60; 20, 50, and 70; 20, 60, and 80; 30, 60, and 80; 30, 60, and 90; 40, 60, and 80

a The system sets the recommended target in an automated fashion.

b The system calculates the MIT available to balance between sensitivity and scan speed.

### Data Analysis

Compound Discoverer (v3.2, ThermoFisher Scientific, Waltham, MA) was used to perform data processing including retention time alignment, background removal, compound extraction and classification, compound grouping, chemical formula prediction, and compound annotation using a node-based methodology (Figure S1 and Table S1). The Full MS data was used for peak picking and the ddMS/MS data for identification only. Total number of features, and total number of annotations were considered for the characterization of the influence of each Full MS parameter, while the number of MS/MS counts, number of annotated compounds with MS/MS information and the spectral quality were used to characterize the influence of MS/MS parameters. Following data processing, features were excluded using general filters such as background removal, mass accuracy (delta = ± 0.5 ppm), and MS/MS for preferred ion. Spectral quality was evaluated by matching experimental spectra with MS/MS spectral library. The mzCloud best match score greater than or equal to 70% was used as the cutoff for spectral similarity. Optimum value for the full MS parameters was defined as the value that provided the highest total number of metabolite annotation within a mass accuracy of 5 ppm and 15% relative standard deviation (RSD) of triplicate measurements. On the other hand, the value that gave the greatest number of annotated compounds with MS/MS information and improved MS/MS spectral quality within a mass accuracy of 5 ppm and 15% RSD of triplicate measurements was identified as the optimum value for the MS/MS parameters. RawBeans was used to evaluate fragment intensity and determine the TopN.^30^ Rv4.1.1 and Microsoft excel 2016 were used for plotting and performing statistical analysis. Based on the criteria described by Sumner and his colleagues,^31^ the level of identification reported here is level 2- 4 for the Full scan parameters and level 2 for the MS/MS parameters.

## Results and Discussion

Measured fragment spectra (MS/MS) of chemical ions is commonly generated using tandem mass spectrometry (LC-MS/MS) to help annotate unknown compounds in untargeted metabolomics.^22^ DDA is one of the most often employed methods for the acquisition of MS/MS spectra.^23^ Multiple full scan and MS/MS parameters in a DDA require the user to define their values. Mass resolution, RF lens, MIT, and AGC target value are a few of the full MS scan parameters. Examples of the MS/MS parameters include signal intensity threshold, mass isolation window, number of microscans per MS/MS scan, an AGC target value, collision energy, and dynamic exclusion. The ability to select which of these parameters and value to be used is advantageous for the user but at the same time also create difficulty in designing a DDA experiment due to the variety of parameters and the wide range of possible values for these parameters. In the sections below, we present the effect of different MS and MS/MS parameters on the coverage of metabolites using untargeted metabolomics. Summary of the Optimum values for the investigated mass spectrometric parameters is presented in Figure S2. The study is limited in that the optimization is performed only for the serum matrix on the Exploris 480 Orbitrap mass spectrometer. Additionally, we solely used reversed-phase (RP) chromatography for our LC conditions. Therefore, the instrument parameters might not always apply to different instrument platforms, LC techniques, or sample matrices.

### Resolution

**Figure 1:**
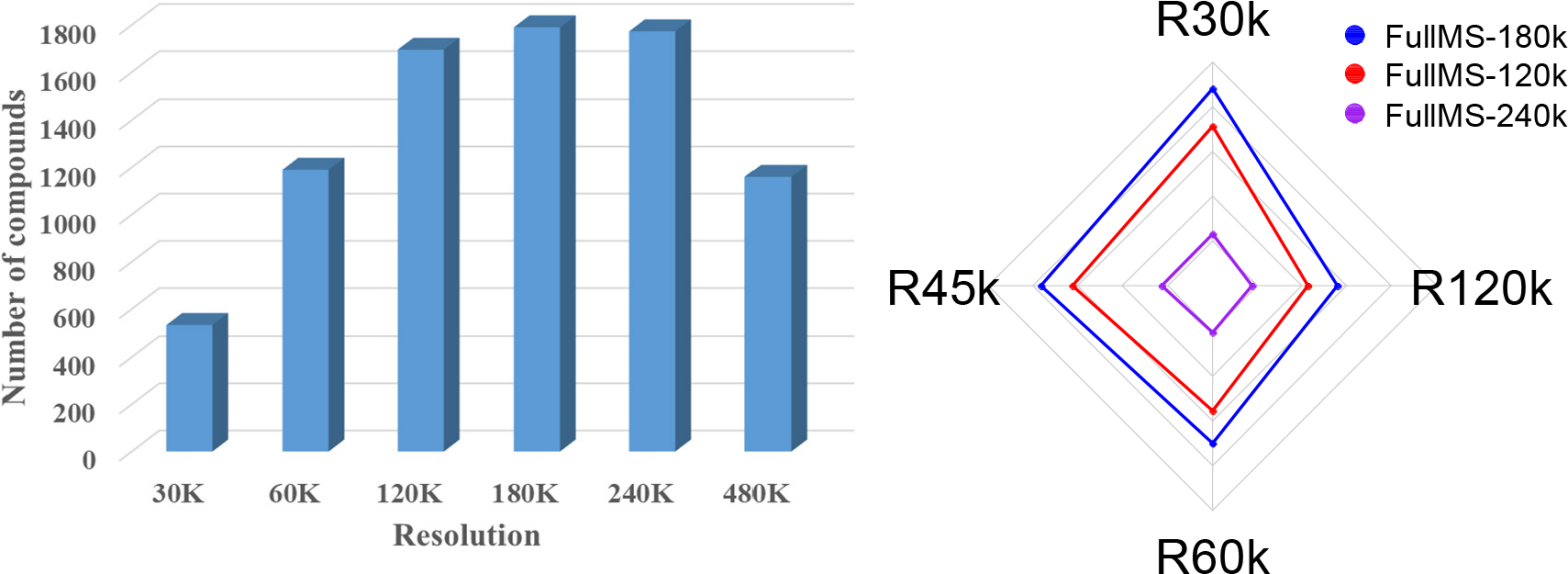
(a) Effect of full MS resolving power on the total compound annotations. (b) Radar plot displaying the effect of MS/MS resolving power on the number of compounds with fragmentation information (MS/MS) at three different full MS resolution settings.

**Figure 2:**
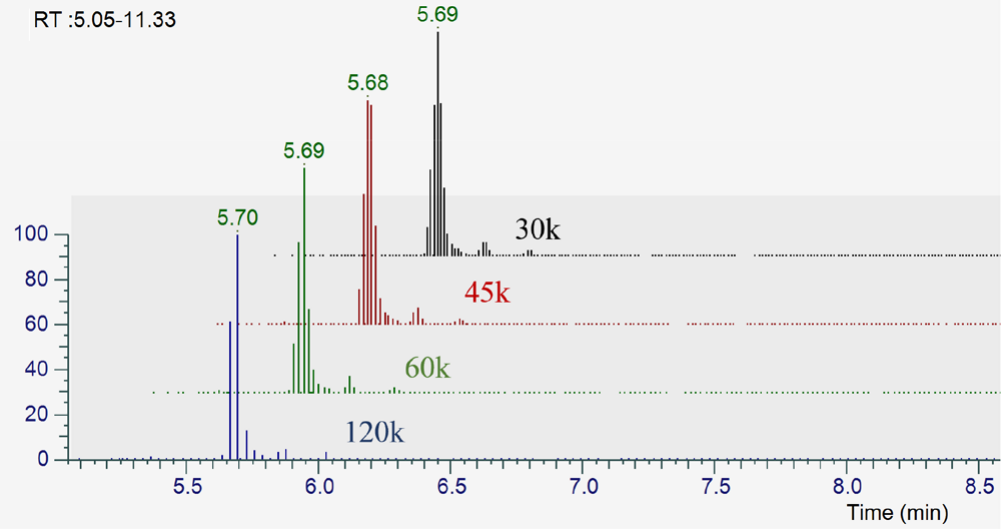
Extracted ion chromatogram displaying the number of data points as a function of the MS/MS resolution.

Generally, high resolution is required to achieve better mass accuracy, enhancing selectivity in complex matrix analysis and, in particular, for the differentiation of isobaric compounds, all of which leads to an increased rate of identification. However, high resolution might also lead to a sensitivity loss due to an increase in the duration of the scan time. Therefore, an ideal balance between the speed and metabolite coverage needs to be established. In this work, the available resolution options ranging from 30k to 480k were evaluated. For the full scan, an increase in the resolution from 30k to 60k turned a similar total number of features, which were 10,225 and 10,687, respectively. Whereas increasing the resolution from 60 to 120k, or 180 or 240k increased total features to 15,287, 17,927 and 18,250, respectively. On the other hand, an increase in the resolution from 30k to 60k increased the number of compounds annotated from 531 to 1190, while changing the resolution from 60k to 120k resulted in annotation of extra 505 compounds. The extra compounds annotated at higher resolution belonged to different classes of compounds such as amino acids, fatty acids, and acyl carnitines. Examples of compounds that were detected at 120, 180, and 240k but not at 30k and 60k included homoserine, creatinine, ornithine, hypoxanthin, indole-3-acetaldehyde, indoleacrylic acid, lauroglycine, oleic acid, hexanoyl carnitine, tiglylcarnitine, succinyl proline, propionyl carnitine, pyrogallol, threosphingosine, *n*-oleoyl-4-aminobutyric acid, glycocyamine, and sorbic acid. The observed increase in the number of annotations in the range of 30–120k could be attributed to two interrelated factors: the decrease in the number of *m*/*z* masked by isobaric matrix interferences (increased selectivity and sensitivity) and improved correct mass assignment (mass accuracy) as the resolution increased. On the other hand, increasing the full MS scan resolution from 180k to either 240k or 480k did not improve the number of annotations considerably. Comparable number of annotated compounds were observed at a resolving power of 120k, 180k, and 240k (Figure 1a). Following the optimization of the full MS resolution, three alternative settings for MS/MS studies were tested (Figure 1b): (1) 120k full MS resolving power and 30, 45, 60, and 120k for MS/MS; (2) 180k full MS resolving power and 30, 45, 60, and 120k for MS/MS; and (3) 240k full MS resolving power and 30, 45, 60, and 120k for MS/MS. In all the three conditions, the number of compounds with MS/MS spectra decreased with increasing the MS/MS resolving power (Figure 1b). The highest number of compounds with MS/MS information was recorded when the full MS scan is performed at a resolution of 180,000 and the MS/MS events were analyzed at a resolution of 30k (Figure 1b). As expected, increase in resolution led to increased cycle time, which resulted in the acquisition of fewer data points on a compound and loss of sensitivity in the MS/MS scans. For example, for a peak that has 10 s width at its base, increasing the resolution from 30k to 120k resulted in declining the number of average data points from 13 to 4 (Figure 2). Overall, the number of metabolite annotations was improved by increasing the resolution (up to 180k) at the MS1 level rather than at the MS/MS level. This implies that improving resolution at the MS/MS level has a minimal impact on the number of metabolite annotations. Taking into account all of these findings, the remaining optimization tests were carried out at a full MS resolution of 180k and an MS/MS resolution of 30k.

It is commonly acknowledged that the low metabolite coverage can be increased by performing an iterative DDA.^21^ To this end, the optimization of the MS/MS resolution (30–120k) was repeated by injecting four sequential injections for deep scanning with the help of AcquireX (Thermo Fisher Scientific, San Jose, US). The number of compounds annotated remained comparable across the investigated range of MS/MS resolution (data not shown), implying that the effect of MS/MS resolution is insignificant when iterative DDA is used.

### RF Lens

**Figure 3:**
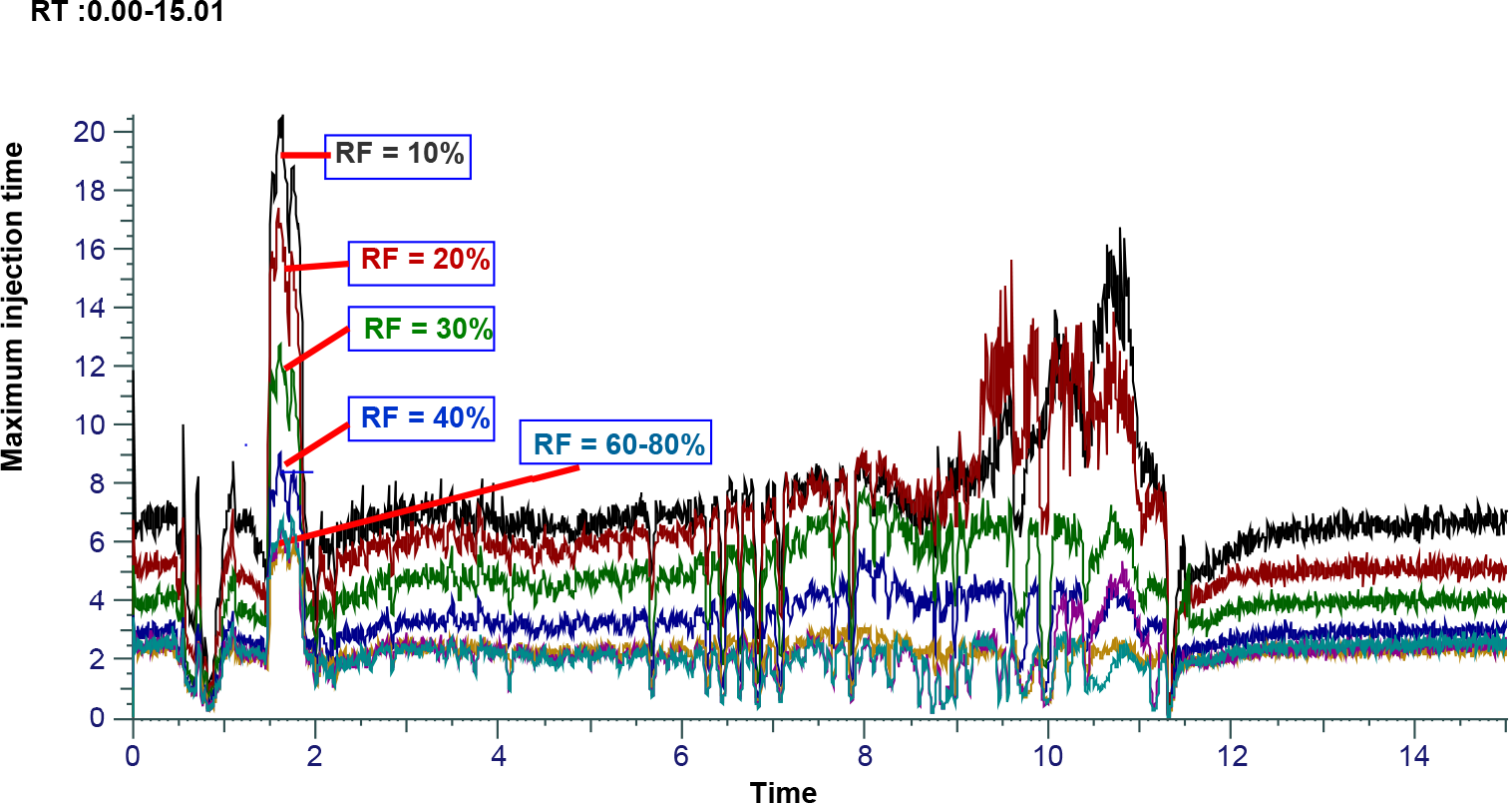
Effect of RF level on the maximum ion injection time (MIT).

The electrodynamic ion funnel is a radio frequency (RF) device that efficiently captures and focuses the ions into a tight beam without needing a DC gradient to propel them forward. By altering the level of the RF lens, significant changes in the sensitivity and consequently the overall number of chemicals discovered were observed (Figure S3a). While a comparable median peak area was reported in the range between 60 and 100% RF level, the median peak area rose as the RF level was varied from 10 to 60% (Figure S3b). On the other hand, the total number of features increased from 10,008 to 18,540 when the RF level was increased from 10% to 60%, while the total of number of features increased from 18,540 to 25,819, 25,501, and 25,200 when the RF level was changed to either 70, 80 or 90%, respectively. Similarly, an increase in the number of annotated compounds was observed between RF levels of 10 and 70%, with a comparable number between 70 and 100%. Increasing the RF level from 10 to 70% increased the number of annotations by about 2.5-fold, while the number of compounds annotated at 70% RF level is 1.5 times higher than that of 60% RF level. The increase in the number of annotated compounds could be partly attributed to the decrease in the maximum ion injection as the RF level increases (Figure 3). Increasing the RF level beyond 70% did not result in a significant increase in the number of annotated metabolites and total features. Noteworthy, very high RF levels might be associated with mass discrimination and/or in source ion fragmentation, which both may result in the loss of sensitivity. An RF level of 70% was, therefore, found to be optimum.

### Mass Isolation Width

**Figure 4:**
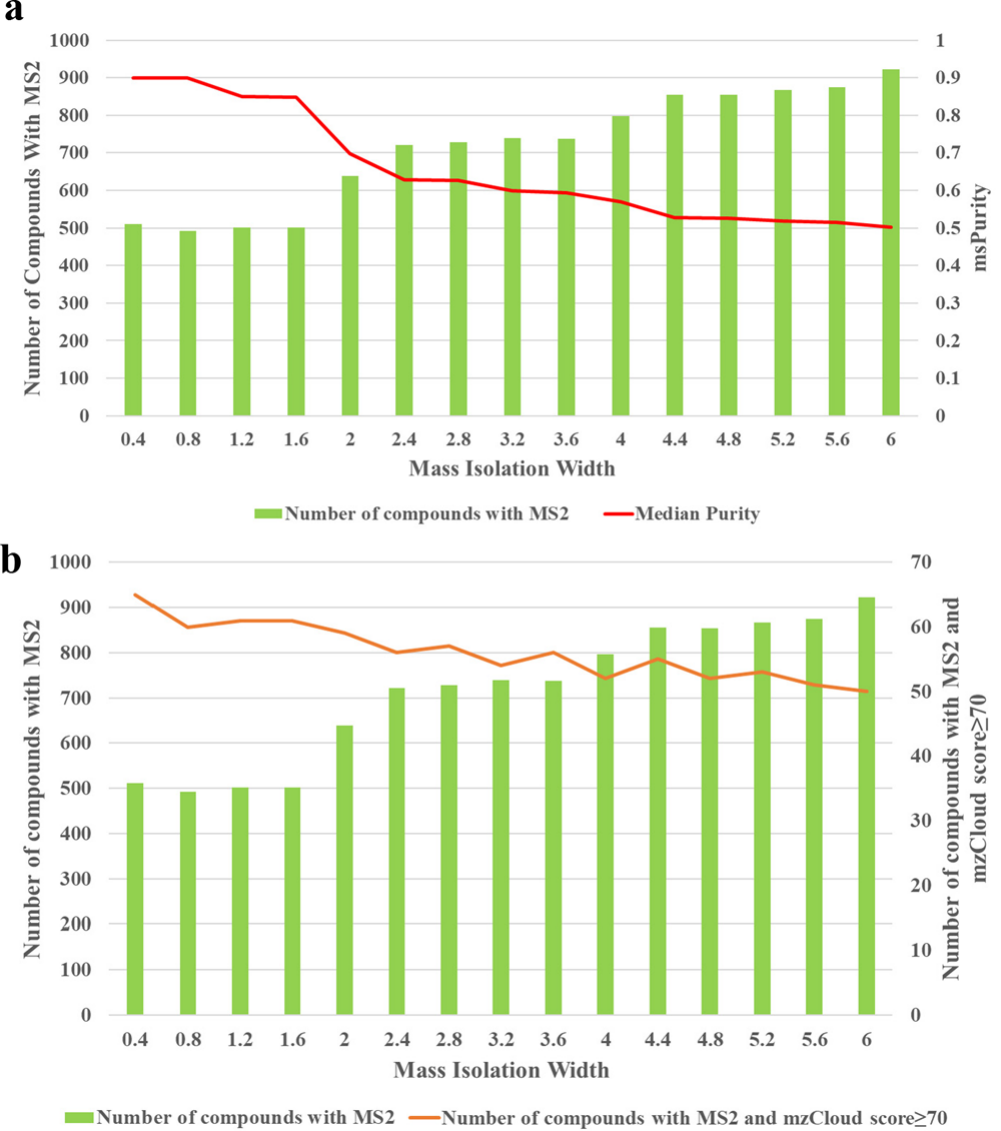
Effect of the mass isolation window on the number of MS/MS spectra acquired: precursor ion purity (a) and spectral score (b).

In the DDA mode, the isolation width (IW), which permits only the precursors within the *m*/*z* values to pass through, is used by the quadrupole to pick metabolite features for the MS/MS scan. MS/MS spectra acquired using wide IW contain isotopologues information, which is known to have an impact on the assignment of molecular formula as the spectral accuracy is an essential factor for the elemental composition determination.^32^ However, as the metabolite mixture derived from the whole metabolome is complex, other metabolites may be coeluted and fragmented into the MS/MS spectrum compromising the purity of the spectrum and reducing the selectivity and spectral matching.^33^ On the other hand, narrower IW provides better selectivity but slightly lower sensitivity. Therefore, spectral information, sensitivity and selectivity all have trade-offs that should be taken in to account when setting the IW.^26^ The effect of the IW on the metabolite coverage was investigated in the range of 0.4–6.0 *m*/*z* (Table 1). A precursor ion purity was calculated for each MS/MS spectra recorded at the different IW using msPurity R package.^34^ The precursor ion purity metric is calculated as a ratio of a selected precursor ion intensity to the total intensity in the isolation window and ranges between 0 and 1.^34^ Values closer to 1 show that the resulting spectra is from a single precursor ion, while values closer to 0 represent the target precursor ion has made little to no contribution from to the total intensity in the isolation window. While the number of compounds with MS/MS spectra increased as a function of the IW in the range between 2.0 and 6.0 *m*/*z*, fewer but comparable MS/MS spectra were acquired when the IW was too narrow (0.4–1.6 *m*/*z*). The median precursor ion purity ranged 0.70–0.90 when the IW was set between 0.4 and 2.0 *m*/*z* (Figure 4). On the other hand, the precursor ion purity reduced considerably (0.60 to 0.50) when an IW of 2.4 *m*/*z* and higher was used (Figure 4a). The precursor ion purity reported here are consistent with the range of precursor ion purity reported in the field of proteomics.^35^ Additionally, the spectral score decreased as a result of increasing the IW. This was confirmed by the decrease in the number of compounds when the mzCloud best match score, one of the scoring systems in the mzCloud spectral library, was applied as a filter to screen for the effect of the IW on the overall spectral quality (Figure 4b). The mass IW of 2.0 *m*/*z* was chosen as the optimum IW that showed relatively higher number of compounds with MS/MS spectra compared to the lower end of the IW (0.4–1.6 *m*/*z*) while maintaining an acceptable level of precursor ion purity (0.70) compared to the higher range of the IW (greater than 2.4 *m*/*z*). However, it is worth mentioning that wide isolation window, used in data independent acquisition (DIA), can be useful to expand the coverage of MS/MS by fragmenting numerous ions simultaneously, particularly for low intensity compounds, and then effectively deconvoluting the chimeric MS/MS spectra computationally.

### Signal Intensity Threshold

This parameter indicates the minimum ion intensity necessary to automatically trigger a fragmentation on a precursor ion in the Full MS scan during the DDA mode of data acquisition. High signal threshold results in lower number of acquired MS/MS spectra but increase the MS/MS spectral overall quality.^26^ On the other hand, lowering the signal intensity threshold is followed by higher number of MS/MS scans, even though the MS/MS spectra may be derived from low intensity ions or chemical noise which compromises the identification.^36^ The effect of signal intensity threshold level from 1e^3^ to 1e^8^ units (Table 1) on the number of acquired MS/MS spectra was examined to determine the optimal value for maximizing metabolite coverage. Generally, the number of acquired MS/MS spectra decreased with an increase in the signal intensity threshold value. However, the number of collected MS/MS spectra stays within the same range when the analysis is restricted to the threshold values that are close to the noise level (1e^3^ to 1e^5^). This could be a sign that the instrument fails to distinguish a metabolite precursor ion from chemical noise, leading to MS/MS data acquisition on a precursor from the chemical noise. On the other hand, with each 10-fold rise in the signal intensity threshold, the number of MS/MS obtained as well as the total number of annotated metabolites are sharply reduced when the intensity threshold is set above 1e^5^ (Figure S4). One other effect of the intensity threshold is the quality of the MS/MS spectra acquired. Setting the intensity threshold at a very low level (at 1e^3^ in our case) might lead to the acquisition of low quality spectra derived from chemical noise or low abundant metabolite precursor ions. Low quality compound spectra are difficult to annotate as most of the software are not capable of handling compound spectra with lower signal-to-noise ratio and hence provides no additional benefit in increasing the number of annotations. Taking the quality of the acquired spectra and number of compounds annotated into consideration, we found the signal intensity threshold of 1e^4^ to be optimum and was used for the rest of the experiments. Defossez and colleagues reported using a threshold 5–10 times lower than the highest signal in the background noise in their DDA method.^26^

### Number of Data Dependent MS/MS Scans (TopN) vs Cycle Time (Top Speed)

**Figure 5:**
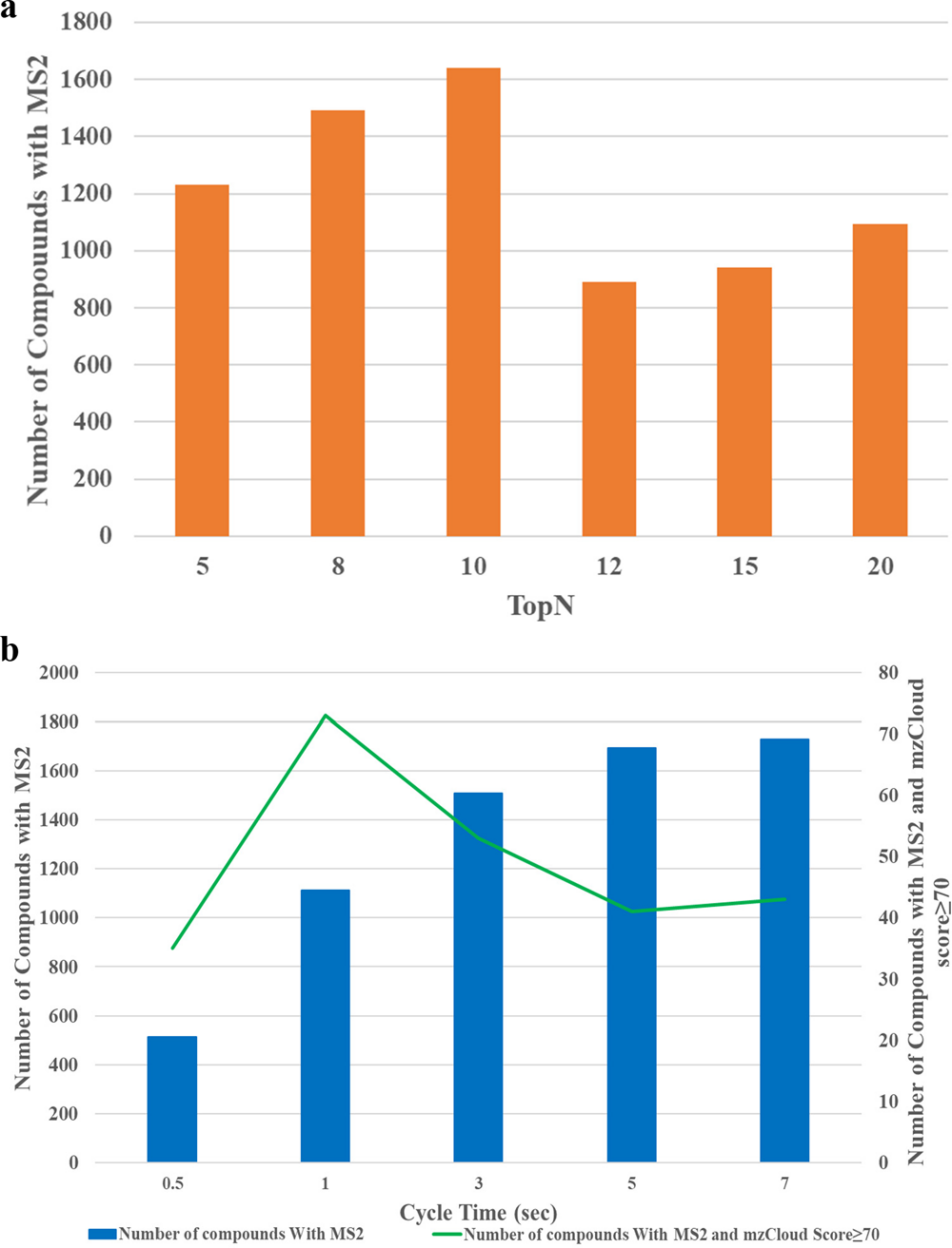
Effect of number of data dependent sans (a) and cycle time (b) on the number of compounds with acquired MS/MS spectra

TopN is among the parameters that can be defined by the user in Orbitrap instruments during data acquisition using the DDA mode. Generally, increasing the number of data dependent MS/MS scans allows more precursor ions from the full MS scan to be picked for fragmentation, though the total number of MS/MS events performed also relies on the duration of the scan cycle and the number of precursor ions in the full MS scan that meet the minimum signal intensity threshold. On the other hand, when the cycle time is used as the data dependent mode, the instrument acquires as many dependent cycles as possible within the specified cycle time (Top Speed) before continuing on to the next experiment. The cycle time determines the number of data points per chromatographic peak. A shorter cycle time allows high peak sampling but fewer MS/MS spectra and vice versa. Figure 5 presents the findings from the analysis of the TopN and Top Speed effects on the number of annotations. In the TopN experiment, 5 to 10 MS/MS dependent scans outperformed the 12 to 20 MS/MS dependent scans. The highest number of annotated compounds was observed with the TopN set at 10 MS/MS events (Figure 5a). When the cycle time was utilized as a data dependent mode, increasing the cycle duration led to an increase in the number of compounds with MS/MS spectra. The number of triggered MS/MS spectra at a cycle time of 1 s was 6412, while it increased to 9781 at a cycle time of 10 s. However, the quality of the acquired MS/MS spectra appeared to decline as the cycle time increased, which, in turn, resulted in a lower score for the compounds in the utilized annotation database (mzCloud) (Figure 5b). This is due to the fact that there is a low peak sampling with numerous MS/MS scans at a higher cycle time and high peak sampling with few MS/MS scans at a lower cycle time. For example, 11 data points per peak were generated on average at a cycle time of 0.5 s, while 6 and 2 data points per peak were recorded on average at a cycle time 1 and 3, respectively. To this end, 1 s would be optimal if cycle time was to be used as a data dependent mode.

However, a higher number of compounds with MS/MS information was observed when Top10 was used as the data dependent mode than when a cycle time of 0.5 or 1 s is used. MS/MS spectra of two (Top 2) and eight (Top 8) compounds can be obtained using the 1 and 0.5 s cycle time method, respectively (Figure S5a and b). Furthermore, comparable experimental cycle times were achieved using the Top 10 and 1 s cycle time methods, which were 1.28 and 1.12 s, respectively (Figure S5c and d). Collectively, greater number of MS/MS spectra can be acquired using the Top 10 (7002 MS/MS spectra) method at a comparable cycle time to that of 1 s cycle time (6412 MS/MS spectra), increasing the overall number of annotated compounds. As a result, for the remaining optimization tests, the Top10 was employed as the data dependent mode. Our result differ from those of Mullard and colleagues, who recommended the top 5 to be used for collision ion dissociation (CID) detection in the Linear ion trap (LIT) as well as CID and higher energy collisional dissociation (HCD) detection in the Orbitrap mass analyzer.^37^

### AGC Target Value and MIT

The automatic gain control (AGC) enables to have more defined number of ions in the Orbitrap by automatically regulating the flux of ions transmitted from the ion source of the instrument.^38^ MIT is the maximum time that it takes to fill the C-trap before being transferred to the Orbitrap mass analyzer, provided that the AGC target value is not already attained. Once the injection time (IT) is reached, the ions will be injected into the Orbitrap, even if the AGC target value is not attained. While the MIT enables the regulation of the ion injection time for species of higher concentration, the use of AGC permits the MIT to be set for ions of low abundance. This ensures optimum mass accuracy and sensitivity for samples with a wider range of concentrations. The AGC and MIT are not independent parameters, and optimization needs to be performed to determine which combination allows higher metabolite coverage. Two factor interaction on the total compound annotation as well as the number of MS/MS scans acquired were therefore explored by testing several combination of AGC and MIT values (Table 1) for both full MS scan and MS/MS experiments.

**Figure 6:**
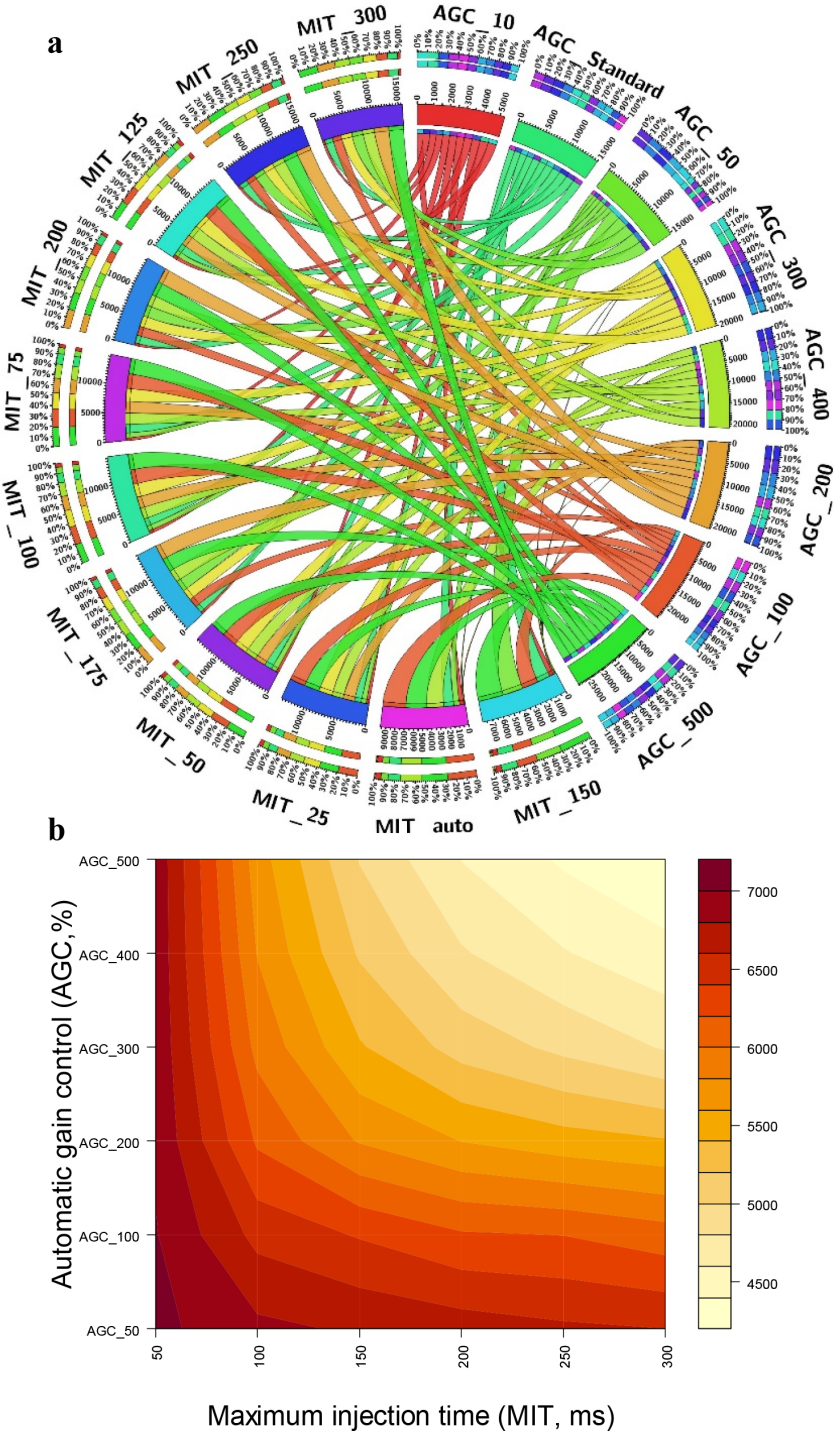
(a) Circos plot displaying the combined effect of AGC target value and MIT on the number of annotations (thickness of each line is proportional to the number of compounds annotated). (b) Contour plots displaying the total number of MS/MS scans as a function of AGC target value and MIT.

Figure 6 presents the interaction of AGC and MIT and their combined effect on the annotation number for both the full MS scan and the MS/MS scan. For full MS scan, the combination of AGC target values at 100% or more and MIT values in the range of 25–125 ms were correlated with higher total number of features, which all were in the range of 12,700–20,469 features. Similar observations were recorded for the total number of annotated compounds, which ranged from 1900 to 2850 metabolites (Figure 6a). AGC target values of 10% and 50% turned the lowest annotated number of metabolites at all MIT values. Increasing the ion injection times beyond 125 ms did not demonstrate a significant increase in total number of features or metabolite coverage compared to the lower MIT values. The highest number of features and total annotations was achieved by combining an AGC target value of 500% (an ion population of 5 x10^6^) and an MIT value of 100 ms and hence was used for the remainder of the experiments. For the MS/MS, comparable results were obtained for AGC target values 50% (5 × 10^4^ ion population) to 500% (5 × 10^5^ ion population) at 50 ms MIT. In all of the tested AGC target values, increasing the MIT beyond 50 ms did not provide an extra advantage and overall resulted in a lower number of annotated compounds (Figure S6). This could be attributed to the decrease in scan rate (long duty cycle) associated with the longer MIT, which in turn results in a lower number of acquired MS/MS events (Figure 6b). The number of MS/MS spectra lowered from 7052 at AGC 50% combined with MIT 50% to 4779 at AGC 300% combined with MIT 300% (Figure 6b). Noteworthy, ion injection times are significantly influenced by electrospray conditions; thus, improving and maintaining stable electrospray conditions should shorten the actual MS and MS/MS injection times.

### Number of Microscans per MS/MS Scan

The effect of the number of averaged microscans per MS/MS scans was examined at 1–5 microscans. Acquiring one microscan per MS/MS event resulted in a noticeably higher number of total features and metabolite annotations for both Full MS scans. Increasing the number of microscans from 1 to 5 led to reduction of total features from 12,639 to 5200. Similarly, increasing the microscan from one to three or five led to 26% and 61% decreases, respectively, in the total number of annotated compounds as well as to 61% and 80% loss, respectively, in the number of compounds with MS/MS spectra. Similarly, the number of compounds with MS/MS spectra decreased by 35% and 59% by increasing the number of microscans from one to three or five, respectively, during the MS/MS spectra acquisition. Although increasing the number of microscans is advantageous for improving the signal-to-noise ratio of low abundant compounds, it generally results in the collection of less MS/MS spectra. This is due to the longer scan and cycle times, which result in a lower number of compounds annotated. Indeed, the average scan time increased to 1.320 and 2.20 s for three and five microscans, respectively, in contrast to 0.440 s with one microscan per full MS scan. Furthermore, the average cycle time increased from about 0.80 s with one microscan per MS/MS scan to 1.50 s with three microscans and 2.20 s with five microscans per MS/MS scan. Therefore, for increased metabolite annotation, one microscan per MS/MS scan was found to be optimal. This is in agreement with the results reported by Kalli and his co-worker for protein identification rates.^39^

### Collision Energy (CE)

**Figure 7:**
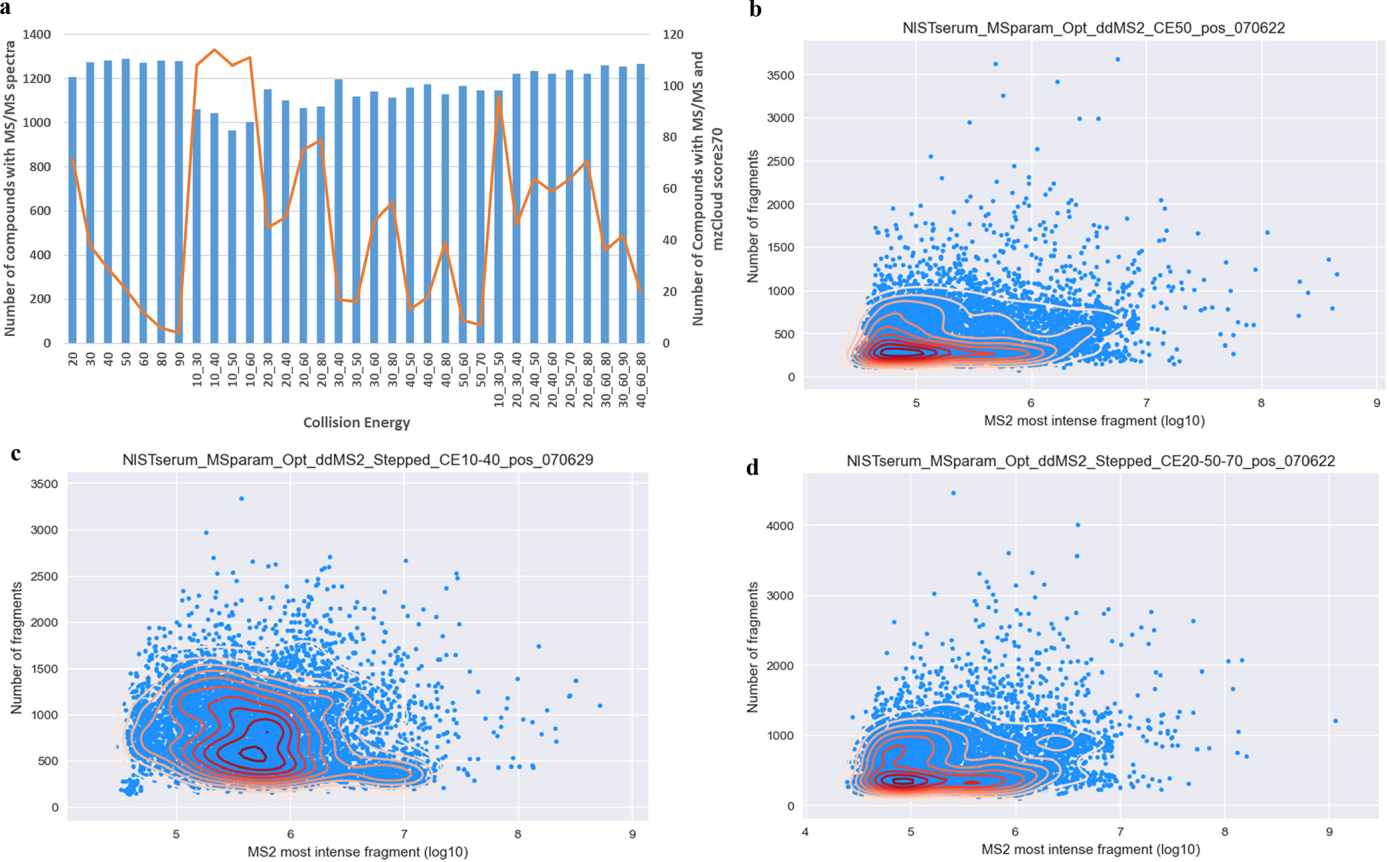
Effect of collision energy on compound annotation and spectral quality (a) and intensity of the most intense fragment ion (b–d).

The fragmentation of ions can vary depending on the CE, so careful optimization is frequently required, because the CE value has a significant potential to affect the MS/MS fragmentation patterns. The quality of the MS/MS fragmentation directly affects the ability to identify metabolites. Overall, 33 different collision energies were tested, recording the energy dependence at fixed CE and stepped CE (step of two and three collision energies) (Figure 7a). Generally, the number of annotated compounds were comparable at the range of CEs investigated, which ranged from 963 at stepped two collision energy of 10 and 50 V to 1288 at fixed collision energy of 50 V. However, there was a noticeable difference in MS/MS spectrum quality, which had an impact on the score supplied by spectral databases like mzCloud. The quality of the fragmentation spectra was diminished at high collision energy, whether fixed or stepped, which decreased the confidence in the results. In comparison, better fragmentation quality was recorded when a step of two collision energies was used. Particularly, stepped collision energies at 10 and 30, 10 and 40, 10 and 50, and 10 and 60 turned the highest quality as revealed by the mzCloud score (Figure 7a). In agreement with the improvement in spectral score, the fragment ion intensities were also better when a step of two collision energies was used than when either fixed or a step of three collision energies was used (Figure 7b–d). Each point on the MS/MS intensity plot (Figure 7b–d) represents a single MS/MS event, where the *y*-axis represents the number of fragment ions in the MS/MS spectrum and the *x*-axis represents the intensity (log transformed) of the most intense fragment ion. Considerable difference can be observed on the *x*- and *y*-axis of Figure 7c, which shows a higher number of fragments in each MS/MS scan and a shift in the density (yellow to red color) of the most intense fragment to higher intensities. Taking all these observations, a step of two collision energies at 10 and 40 V was used for testing the rest of the mass spectrometer parameters. Previous research suggested using three activation energies (low, medium, and high) to increase the possibility of acquiring MS/MS mass spectra related to significant precursor ion fragmentation suitable for efficient metabolite annotation.^37^

### Dynamic Exclusion

After the mass spectrometer has gathered enough information about an ion, it can be prevented from triggering more data-dependent scans using a technique called dynamic exclusion. The most intense peaks in a mass spectrum are purposefully ignored using dynamic exclusion so that data from the lower intensity peaks can be collected. For the dynamic exclusion parameter optimization, the following settings were used: repeat count 1 and exclusion duration 3, 5, 7, 10, 15, 20, 40, 60, 80, and 100 s; repeat count 2; repeat duration 30 s, and exclusion duration 20, 40, 60, 80, and 100 s. With a repeat count of 1, the number of compounds with MS/MS spectra increased as the duration exclusion increases until 40 s but remained comparable in the range between 40 and 100 s. However, an increase in the number of compound fragmentation was followed by a decrease in spectral quality, particularly at dynamic exclusion duration 15 s and higher. This could be due to, at higher exclusion duration, compounds that might be fragmented only once, leading to lower signal-to-noise ratio of fragment ions or due to the MS/MS triggered far from the apex.^40^ On the other hand, when a repeat count of 2 and repetition duration of 30 s were utilized, the influence of exclusion duration on the coverage of MS/MS scans was relatively smaller and generally lower than the number recorded with a repeat count of 1 at the same exclusion duration. As a result, the optimal dynamic exclusion under the current experimental set up was found to be 10 s duration and a repeat count of 1. Previous studies have reported much higher exclusion durations.^25,41^ Note that the choice of optimal dynamic exclusion duration is significantly influenced by the chromatographic conditions used to run the samples.^41^

## Conclusion

For untargeted metabolomics to be successful in producing high quality data suited for hypothesis exploration in physiological systems, the performance and optimization of the LC and MS systems are essential. In summary, the quality of the collected MS and/or MS/MS spectra and consequently the identification of the metabolites heavily depended on resolution, signal intensity threshold, RF lens, MIT, AGC, TopN, cycle time, microscan, and mass isolation window. In comparison to other instrument settings, dynamic exclusion and collision energy had the least impact on the total number of annotated compounds across the investigated range of values. However, the fragment spectral quality that is related to the annotation confidence still depends on the optimized collision energy and dynamic exclusion. The findings of this work offer information that can be used to understand and improve mass spectrometric parameters for untargeted metabolomics.

The study is limited in that the optimization is performed only for serum matrix on the Exploris 480 Orbitrap mass spectrometer. In addition, our LC conditions were based on only reversed phase (RP) chromatography. The instrument settings may not therefore necessarily translate across different instrument platforms, LC methods, or sample matrices; however, the general logic applied and workflow for optimization would be appropriate to consider at the onset of any new set up for untargeted metabolomics.

## Supporting Information Available

Compound Discoverer 3.2.0 workflow tree; general parameter and settings for each compound Discoverer 3.2.0 node used; summary of optimum values; effect of % RF lens; effect of signal intensity threshold; number of data dependent scans (TopN); experimental cycle time (PDF)
