# Supplementary material for: Optimization of Mass Spectrometric Parameters in Data Dependent Acquisition for Untargeted Metabolomics on the Basis of Putative Assignments
Source: J Am Soc Mass Spectrom. 2023 Jul 7;34(8):1621–31. doi: 10.1021/jasms.3c00084 (PMC10402710; doi:10.1021/jasms.3c00084)
Supplement: Supplementary file 1 — js3c00084_si_001.pdf [file js3c00084_si_001.pdf]

Supporting information for

Optimization of mass spectrometric parameters in data dependent acquisition for untargeted metabolomics on the basis of putative assignments

Hailemariam Abrha Assress[1, 2], Mario G. Ferruzzi[1, 2], Renny S. Lan*[1, 2]

[1]Arkansas Children's Nutrition Center, Little Rock, Arkansas, 72202

[2]Department of Pediatrics, University of Arkansas for Medical Sciences, Little Rock, Arkansas, 72205

*Slan@uams.edu

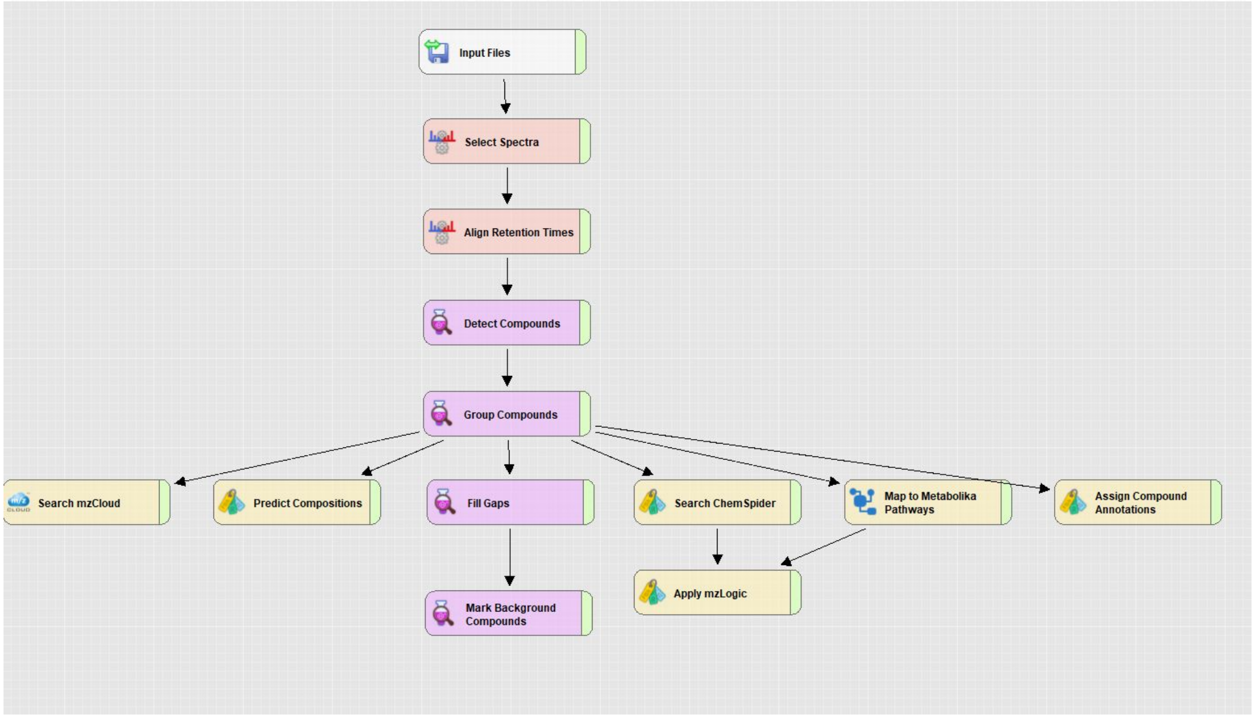

Figure S1: Workflow tree from Compound Discoverer 3.2.0 software displaying data processing nodes and the associated workflow connections. Nodes for initial data processing such as Input Files, Select Spectra nodes, and Align Retention times are included. The Detect Compounds node is used to implement peak detection for unknowns. The Group Compounds node integrates information from multiple raw data files. The Predict compositions, Search mzCloud, Search ChemSpider, and Apply mzLogic nodes were utilized as compound annotation nodes Based on both MS1 and MS2 spectrum data. The Assign Compound Annotations node was used to prioritize the annotation source. Lastly, the Mark Background Compounds node was used to indicate compounds arising from the experimental blank.

Table S1: Description of the general parameter and their settings used for each data processing node during data processing using the compound Discoverer 3.2.0

**Node**	**General parameter and settings**
Input files	Raw data were imported
Select spectra	Precursor Selection: Use MS(n - 1) Precursor<br>Use Isotope Pattern in Precursor Reevaluation: True<br>Provide Profile Spectra: Automatic<br>Store Chromatograms: False<br>Lower RT limit: 0<br>Upper RT limit: 15<br>Total intensity threshold:<br>Minimum peak count:<br>Min. precursor mass: 50 Da<br>Max. precursor mass: 1000 Da<br>Scan type: any<br>Min. Collision Energy: 0
Align Retention Times	Alignment Model: Adaptive curve<br>Alignment Fallback: Use Linear Model<br>Maximum Shift [min]: 2<br>Shift Reference File: True<br>Mass Tolerance: 5 ppm<br>Remove Outlier: True
Detect Compounds	Mass Tolerance [ppm]: 5 ppm<br>Intensity Tolerance [%]: 30<br>S/N Threshold: 3<br>Min. Peak Intensity: 50,000<br>Ions: [2M+H]+1; [2M-H]-1; [M+2H]+2; [M+H]+1; [M+H+Na]+2; [M+H-H2O]+1; [M+Na]+1; [M-2H]-2;; [M-H]-1; [M-H-H2O]-1<br>Base Ions: [M+H]+1; [M-H]-1<br>Min. Element Counts: C H<br>Max. Element Counts: C90 H190 Br3 Cl4 K2 N10 Na2 O18 P5 S5<br>Filter Peaks: True<br>Max. Peak Width [min]: 1<br>Remove Singlets: True<br>Min. Scans per Peak: 6<br>Min.Isotopes:2
Group Compounds	Mass Tolerance: 5 ppm<br>RT Tolerance [min]: 0.2<br>Preferred Ions: [M+H]+1; [M-H]-1
Fill gaps	Mass Tolerance: 5 ppm<br>S/N Threshold: 1.5

	Use Real Peak Detection: True
Mark Background Compounds	Max. Sample/Blank: 5<br>Max. Blank/Sample: 0<br>Hide Background: True
Search mzCloud	Compound Class: all<br>Precursor Mass Tolerance: 10 ppm<br>FT fragment Mass Tolerance: 10 ppm<br>Library: Autoprocessed; Reference<br>Max. # Results:10<br>Identity search: Cosine<br>Match activation type: True<br>Activation energy tolerance:20
Predict Compositions	Mass Tolerance: 5 ppm<br>Min. Element Counts: C H<br>Max. Element Counts: C90 H190 Br3 Cl4 N10 O18 P5 S5<br>Min. RDBE: 0<br>Max. RDBE: 40<br>Min. H/C: 0.1<br>Max. H/C: 4<br>Max. Candidates: 10<br>Max. Internal Candidates: 200<br>Intensity Tolerance [%]: 30<br>Intensity Threshold [%]: 0.1<br>S/N Threshold: 3<br>Min. Spectral Fit [%]: 30<br>Min. Pattern Cov. [%]: 90<br>Use Dynamic Recalibration: True<br>Use Fragments Matching: True<br>Mass Tolerance: 5 ppm
Search ChemSpider	Databases(s): BioCyc; Human Metabolome Database; KEGG<br>Search Mode: By Formula or Mass<br>Max. # of results per compound: 10<br>Result Order (for Max. # of results per compound): Order By Reference Count (DESC)<br>Check all predicted Composition: False
Map to Metabolika Pathways	Metabolika Pathways: All<br>Search Mode: By Formula or Mass<br>Mass Tolerance: 5 ppm<br>Max. # of results per compound: 3
Apply mzLogic	FT Fragment Mass Tolerance: 10 ppm<br>IT Fragment Mass Tolerance: 0.4 Da<br>Max.# Compounds: 0

	Max. # mzCloud Similarity Results to consider: 10<br>Match Factor Threshold: 30
Assign Compound Annotations	Mass Tolerance: 5 ppm<br>Data Source 1: mzCloud<br>Data Source 2: ChemSpider<br>Data Source 3: Predicted Compositions<br>Data Source 4: Metabolika Search<br>Use mzLogic: True<br>Use Spectral Distance: True<br>SFit Threshold: 20<br>SFit Range: 20

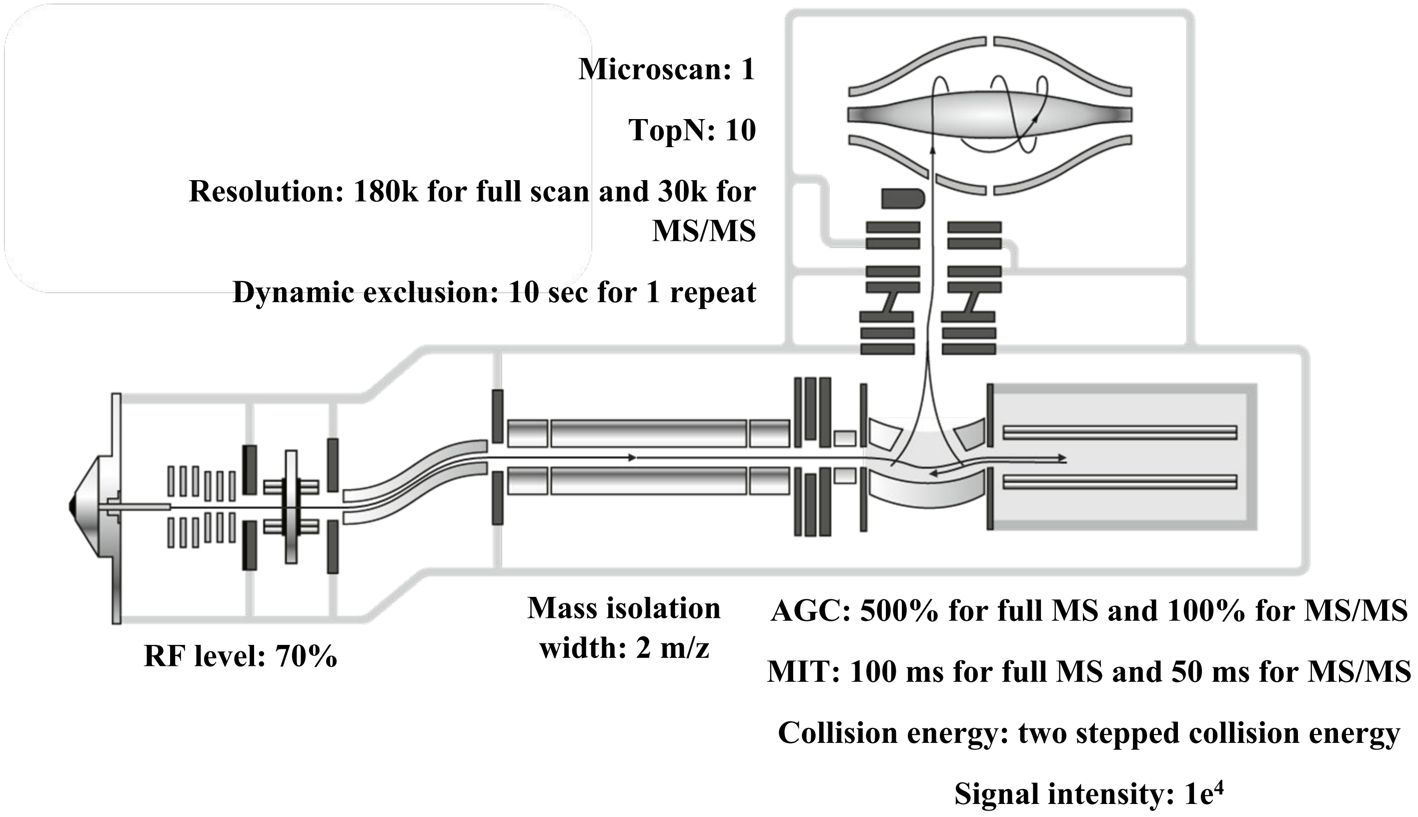

Figure S2: Summary of the Optimum values for the investigated mass spectrometric parameters

a

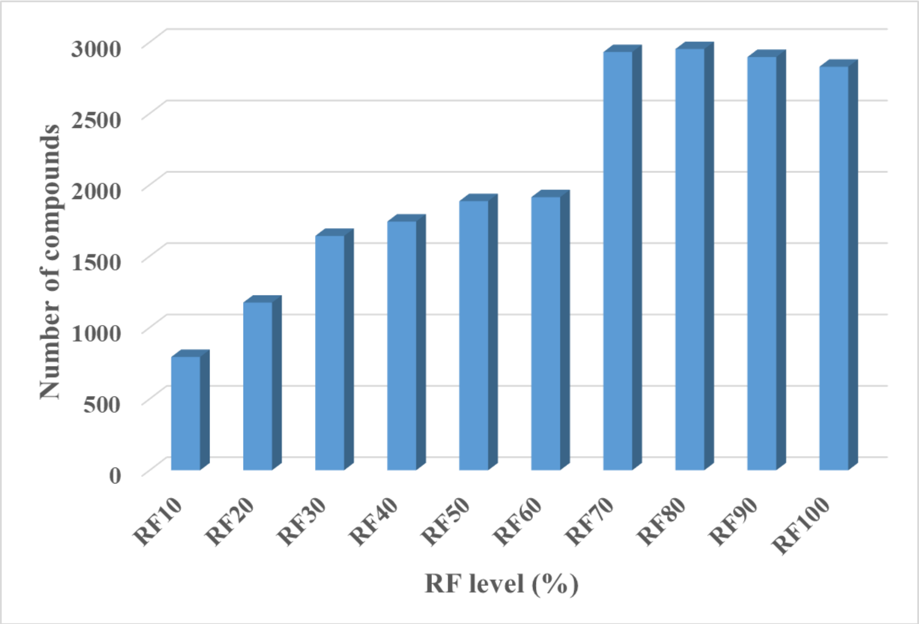

b

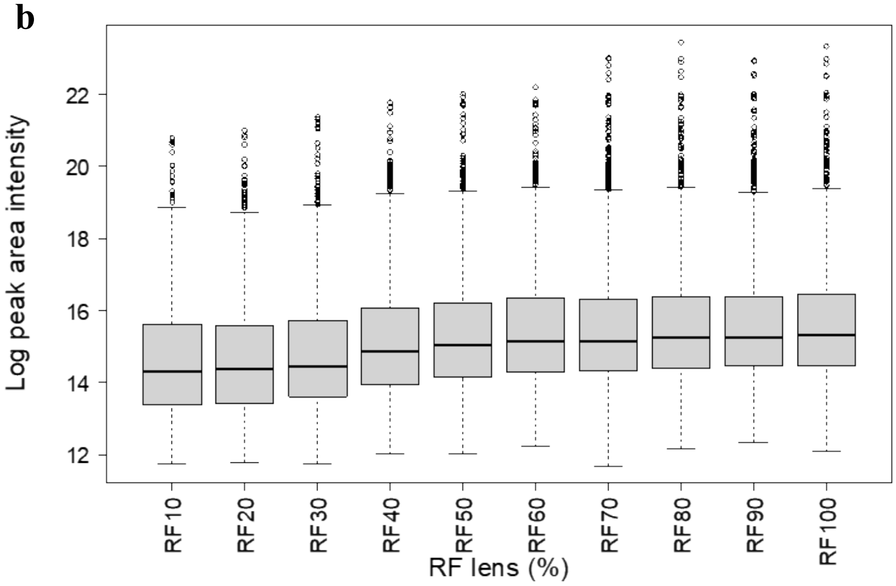

Figure S3: a) Bar graph representation of the effect of % RF lens on the number of compound annotation, (b) boxplot for peak area intensity. The bars in the box plot show the minimum and maximum values, while the line within the box shows the median value.

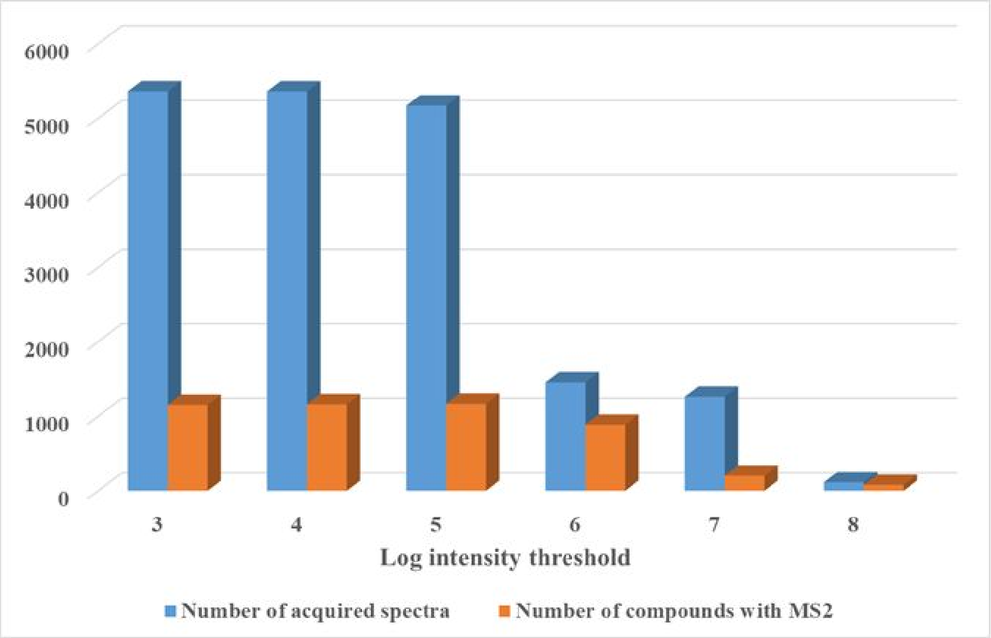

Figure S4: The effect of signal intensity threshold on the number of acquired MS/MS spectra and annotation of metabolites.

a

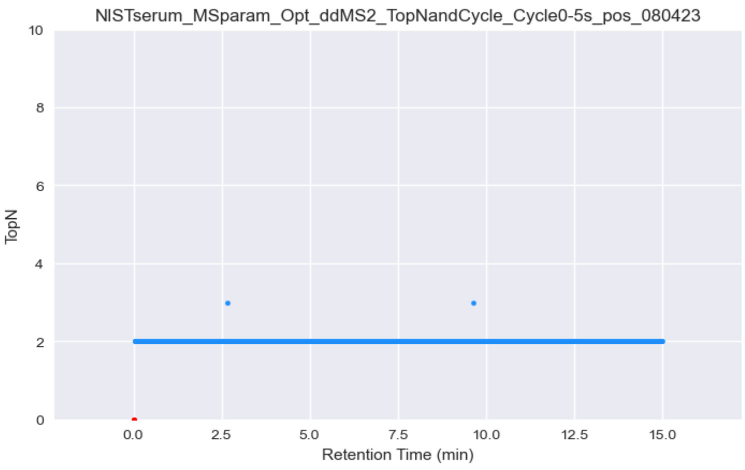
NISTserum_MSparam_Opt_ddMS2_TopNandCycle_Cycle0-5s_pos_080423
TopN
Retention Time (min)
0
2
4
6
8
10
0.0
2.5
5.0
7.5
10.0
12.5
15.0

b

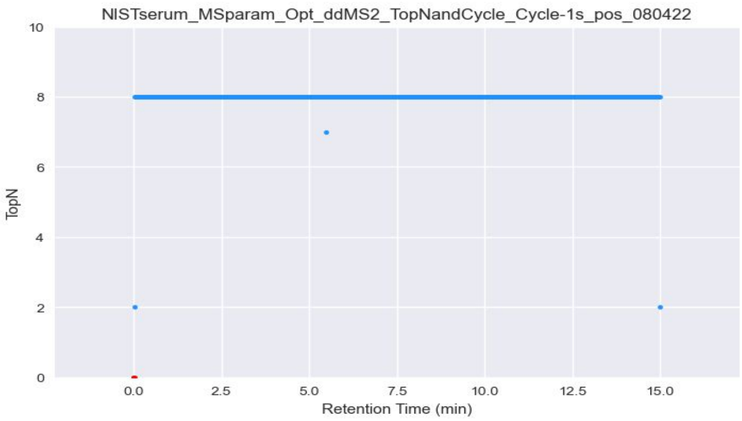
NISTserum_MSparam_Opt_ddMS2_TopNandCycle_Cycle-1s_pos_080422
TopN
Retention Time (min)
0
2
4
6
8
10
0.0
2.5
5.0
7.5
10.0
12.5
15.0

c

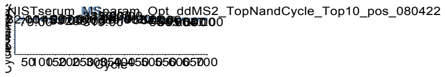
NISTserum_MSparam_Opt_ddMS2_TopNandCycle_Top10_pos_080422
Cycle

d

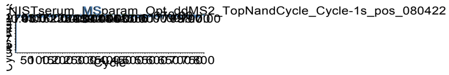
NISTserum_MSparam_Opt_ddMS2_TopNandCycle_Cycle-1s_pos_080422
Cycle

Figure S5: Number of data dependent scans (TopN) performed at 0.5 s cycle time (a) and 1 s cycle time (b). Experimental cycle time when Top10 is used (c) and when a cycle time of 1 s (d) is used.

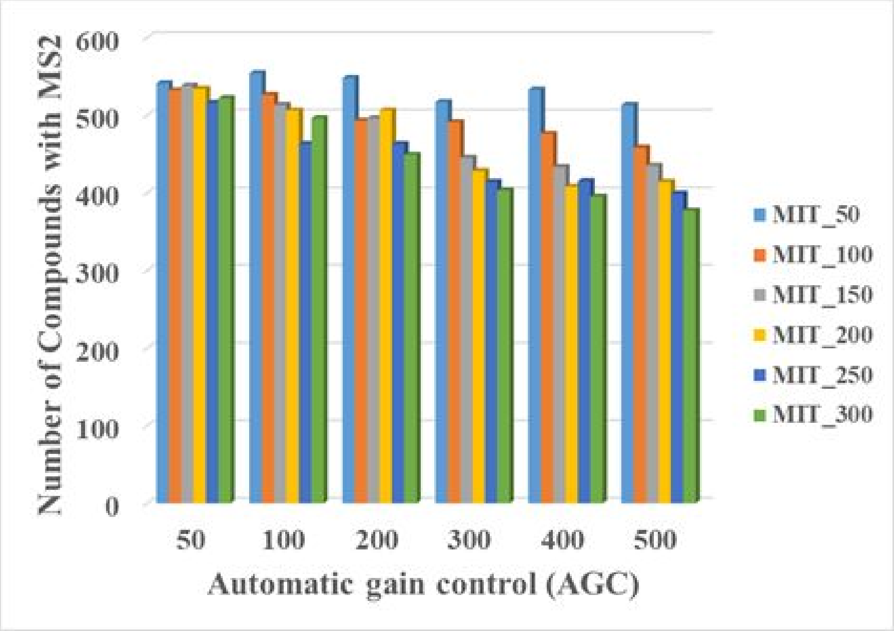

Figure S6: The effect of MIT on the number of acquired MS/MS spectra and compound annotation.
